# Real-Time Extended Interface Automata for Software Testing Cases Generation

**DOI:** 10.1155/2014/731041

**Published:** 2014-04-29

**Authors:** Shunkun Yang, Jiaqi Xu, Tianlong Man, Bin Liu

**Affiliations:** ^1^School of Reliability and Systems Engineering, Beihang University, Beijing 100191, China; ^2^Science & Technology on Reliability & Environmental Engineering Laboratory, Beijing 100191, China

## Abstract

Testing and verification of the interface between software components are particularly important due to the large number of complex interactions, which requires the traditional modeling languages to overcome the existing shortcomings in the aspects of temporal information description and software testing input controlling. This paper presents the real-time extended interface automata (RTEIA) which adds clearer and more detailed temporal information description by the application of time words. We also establish the input interface automaton for every input in order to solve the problems of input controlling and interface covering nimbly when applied in the software testing field. Detailed definitions of the RTEIA and the testing cases generation algorithm are provided in this paper. The feasibility and efficiency of this method have been verified in the testing of one real aircraft braking system.

## 1. Introduction

Interface automata, as a kind of modeling method that describes the interactions between components and the environment, have been applied gradually in many fields in recent years. By using the input assumption and output guarantee, the interface automata express the sequential relationship of the interaction between the system and the environment to describe the operation of the internal system and the action of the environment interface formally. But when it is applied in the software testing field, there are still some inevitable problems including the limited description ability of temporal information, which leads to a lack of accuracy to describe some embedded real-time system, and the neglect of input controlling and interface covering, which result in that some tests which need interface covering find it hard to use this modal conveniently.

Alur and Dill [1] put forward the timed automata method, which bring accurate time description for different state transition actions, and are able to describe the temporal relationship between different state transition actions accurately and formally by introducing the time language. But time automata pay more attention to internal information of the system instead of the interaction between the system and the environment. The timed interface automata are proposed on the basis of interface automata by De Alfaro et al. [2], which can describe the delay time and the keep time of the state transition process by introducing the two new parameters, time guard and clock variables. But there are also disadvantages of the timed interface automata. For some real-time system, the input/output signals may be periodic. However, the timed interface automata cannot provide a formalized description for the constraint and the change of a periodic variable.

Besides, the automata method also has some defects when it is applied to the software testing field. As Raffelt et al. proposed the dynamic testing method based on learning automata, as a result of the automata's learning features, the method was combined with many mature learning algorithms to make dynamic tests. At the same time, an application of web services testing is provided [3]. Its limitation is that the method is focused on network services and internal structure of cellular automata; it may not be suitable for embedded software; Nielsen and Skou proposed a real-time black box conformance testing method for uncertain systems based on timed automata model, and the experimental results show its superiority [4]. But because it is based on the timed automata, the ability of temporal information description of input and output is very limited; a new testing method has been proposed by Liangming et al. based on the interface automata model, it divides state sequences into different sections [5], and the covering algorithm is given. But it focuses on path covering of the automata model, while the interface covering cannot be guaranteed. Thus, when the traditional interface automata method is applied in software testing, especially in the interface testing of real-time system, there are some limitations. That is, the period information of the input and output cannot be described. Besides, it is not convenient to control the input value including its temporal information (especially to automatic control).

To solve the problems above, this paper puts forward the RTEIA on the basis of interface automata and timed automata; its main contributions are as follows.

1. By applying the time words to describe the automaton of input/output action, it provides clearer and more detailed temporal information description for the automata model, which contains both the moment and the period information. With the values and the temporal information of input/output packaged, unified actions and descriptions are facilitated.
2. By establishing input automata for each input interface to construct internal structure of input automata, this method nimbly solved the problems of input control and interface covering when applied to the software testing field.

The structure of the rest of this paper is as follows.  Section 2 focuses on the theory of interface automata and related research background. Specific definition of RTEIA is given in Section 3; Section 4 mainly expounds the test case generation based on this method and presents a generation algorithm which is verified and discussed with an instance. Conclusions and outlooks for future work are in the last section.

## 2. Preliminary

In software testing, usually there is a very complex interaction between the system and the environment. For the convenience of testing modeling and automatically generating test cases, formalized description of the interface is necessary. The following lists some formal languages that describe the interface interaction based on automata theory.

### 2.1. Interface Automata

De Alfaro and Henzinger put forward the interface automata in 2001 [6]. Interface automata's main features are as follows: (1) “positive method” is used to define the interface compatibility issues under different environment rules and (2) the game idea is a term used to describe the process of the problem solving.

Unlike the traditional method which records and gathers input domain and output domain, interface automata also describe the temporal characteristics of the input and output, integrate the input assumption and output guarantee, and provide formalized description. Output guarantee reflects the order of the various external services request from the interface which denotes the assumptions made to the environment. Input assumption reflects the order of the services provided to the external by the interface which denotes a description of the automata's own behavior.

Optimistic method is a kind of compatibility judging processing method of the interface and environmental relationship; the traditional method (or named pessimistic methods) is that as long as there is a kind of external environment to make illegal status be achieved, it considers the combined interface incompatible. But the optimistic method believes that as long as there is a kind of external environment which can make the combined interface function normally, it considers that they are compatible. Its advantage is to make the number of interface states greatly reduce after the combination, which makes the interface automata as a light-weighted formalized system.

Game ideas are as follows. Interface automata finally attribute the operation of the interface and the environment to a game; the environment is to find a strategy to meet all interface input assumptions as far as possible. If environment wins the game, we can consider that interfaces are compatible; otherwise, they are incompatible.

With the developing of the theory, in addition to the main application to verify components compatibility of the modular development mode, interface automata method was applied to a variety of related fields gradually; that is, because interface automata model's description of the state transition is similar to the web service user transferring and link jumping, the interface automata theory was applied to the description of building web services by Li et al. [7], and the relevant verification algorithm was given; Zhang et al. [8] applied the interface automata to analyze the usability of composite web services building, and then they proposed hybrid interface automaton based on the interface automata method. The algorithm used to check accessibility, well-formedness, and compatibility for the network physical system is given as well. Due to the lightweight and generality of interface automata model, Karimpour et al. [9] combined it with the queuing theory and applied it to the modular software system performance prediction field.

### 2.2. Timed Automata

Alur and Dill put forward a kind of automata method that contains temporal information named timed automata [1]. Timed automata are a kind of formalized model based on the finite state automata extended with clock variables to describe the continuous changes of the time and analyze the behavior of the real-time systems. An important properties of this theory lies in its ability to detect the reachability of any state in the timed automata.

Timed automata introduced the time words based on the finite state automata and added clock variables into the time words to make the input and output information contain the temporal information. Timed automata use logical relationship to describe the temporal relationship between different input/output actions by the formalized language. But this method focuses on the internal state transition actions information only and does not take consideration of the interactive actions between the system and the environment.

Bengtsson and Yi did a further study of timed automata on semantics and the implementation algorithm and put forward the timed automata semantic system that can realize the model from the theory to the application. The corresponding algorithm and verification tools were provided as well [10]. Timed automata were applied to the real-time distributed system verification by Wen et al., based on constructing timed automata for controller area network, tasks, and the operating system kernel, respectively, to analyze the system and to verify its validity by experiments [11]. Calder et al. extended the interface automata with another way of temporal information and proposed RTIA real-time interface automata for scenario-based timing specifications [12]. Jin et al. expanded the method of [11] to solve the timed automata modeling problems of complex real-time systems and introduced the hierarchical timed automata (HTA) by partitioning the component level and hiding and expanding execution of corresponding chain to make the description of multilayered components more accurate [13]. Some researches were started in the timed automata model validation and the mutual transformation among different modeling languages, and so forth, [14–17].

### 2.3. Timed Interface Automata

De Alfaro et al. extended the interface automaton method and proposed the timed interface automata [2].

Interface automata method introduces time constraints in order to describe the real-time system better. It adds a clock variable for interface automata; each state has a predicate made of a set of time variables called time condition. The time condition restrained the occurring conditions of the input/output and also introduced the guard which is used to restrain the input/output that shall meet the conditions.

Because the input/output actions themselves are not time-consuming, the state transition does not require any extra time. So if the executions of the input/output were repeated infinitely, the automata time will stop forever, which is obviously inconsistent with the facts. This action is known as Zeno behavior. To this end, the authors put forward a well-formedness criterion which rules that, in any reachable state, time interface automata should guarantee that the time variable of input/output actions would never stop.

In order to apply the timed automata to web services field, based on the concurrent characteristics of web services, Esmaeilsabzali et al. put forward Interface automata with complex actions. By introducing complex behaviors that do not interlace with other components for interface automata, this method can describe the characteristics of the concurrent systems better [18]. In view of the pervasive systems, Calder et al. put forward the pervasive interface automata [12], according to the characteristics of the pervasive system that context is closely linked. They expand the concept of input and output of interface automata theory and introduce the function calling and being called, so that the pervasive interface automata can be described better. Raclet et al. combined the interface automata with modal specification method, summarized the predecessors' combination method, and put forward the modal interface automata [19]. Then, Krka and Medvidovic collated and improved modal interface automata [20].

The methods above are all trying to describe the interaction of the interface including temporal information, but there are still drawbacks. For instance, the timed automata lack the view of the interface and the event description of the timed automata relies on state transition logic relationship. The timed interface automata lack a complete description of period information; besides, the ability of input controlling is scanty. Therefore, this paper presents the RTEIA, which adds clearer and more detailed temporal information description for the automata model. And, by the establishment of input interface automaton for every input interface, it also nimbly solve the input controlling and interfaces covering problems when applied in the test field.

## 3. The Real-Time Extended Interface Automata

To make up for the existing problem of interface automata model such as incomplete description temporal information and the lack of considering of the input controlling, we introduce time words and input automata for interface automata as the RTEIA.

### 3.1. Time Language

Considering the defect of the interface automata when it is used to describe a real-time system, we need a language that can describe the temporal characteristics of the system input and output parameters. Therefore, we introduce and improve the concept of time language from the timed automata [1] to describe the temporal character of the real-time system. Time language contains 3 time parameters; as in the case of the dense-time model, the set of nonnegative real numbers, *R*, is chosen as the time domain. Each input/output *σ* is coupled with two temporal parameters time sequence *τ* and period parameter *λ* as defined below.

**Definition 1**

A time sequence *τ* = *τ*_1_, *τ*_2_,… is an infinite sequence of time values *τ*_*i*_ ∈ *R* with *τ*_*i*_ > 0, satisfying the following constraints.

1. Monotonicity: *τ* increases strictly monotonically; that is, *τ*_*i*_ < *τ*_*i*+1_ for all *i* ≥ 1.
2. Progress: for every *t* ∈ *R*, there is some *i* ≥ 1 such that *τ*_*i*_ > *t*.

**Definition 2**

A period parameter *λ* = *λ*_1_, *λ*_2_,… is an infinite sequence of time values *λ*_*i*_ ∈ *R* with *λ*_*i*_ ≥ 0.

*τ* of an input/output *σ*, *λ* is used to express the period information. A time word over an alphabet Σ is a pair (*σ*, *τ*, *λ*) where *σ* = *σ*_1_ *σ*_2_… is an infinite word over Σ, *τ* is a time sequence, and *λ* denotes the period information. Timed language over Σ is a set of timed words over Σ.

Let one consider some examples of timed languages.

**Example 3**

Let the alphabet be Σ = (*a*, *b*) and its time language *L*_*i*_ → (*σ*, *τ*, *λ*).

To describe: there is no *b* after time 3.2, and its period is 300. Its time language can be as

(1) L1={(σ,τ,λ) ∣   ∀i.  ((τi>3.2)⟶(σi=b,λi=300))}.

To describe:   *a* and *b* are alternate, and, for the successive pairs of *a* and *b*, the time difference between *a* and *b* keeps increasing, and the period keeps decreasing in the same time. Its time language can be as

(2) L2={((ab)ω,τ,λ) ∣    ∀i.  ((τ2i−τ2i−1)<(τ2i+2−τ2i+1),      λ2i>λ2i+2,λ2i−1>λ2i+1)}.

**Figure 1:**
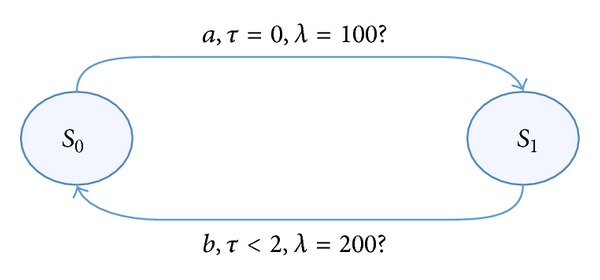
An example of time language.

To describe:   *a* and *b* are alternate, the period of *a* is 100, the period of *b* is 200, and *b* should be later than *a* in 2, which is shown in Figure 1.

Its time language can be as

(3) L3={((ab)ω,τ,λ) ∣   ∀i.  (τ2i<(τ2i−1+2),λ2i=100,      λ2i+1=200)}.

### 3.2. The RTEIA

With the time words as the tool for describing the temporal information, we define the RTEIA based on the interface automata method. The RTEIA packs the temporal information to describe the various actions of the automata clearly.

It is defined as follows.

**Definition 4**

The RTEIA *P* is a quintuple; *P* = 〈*V*_*P*_, *V*_*P*_^init^, *A*_*P*_^*I*^, *A*_*P*_^*O*^, *T*_*P*_〉, where

1. *V*_*P*_ is a set of states;
2. *V*_*P*_^init^⊆*V*_*P*_ is the initial set of states, and *V*_*P*_^init^ contains at least one state; if *V*_*P*_^init^ = *⌀*, one says *P* is empty;
3. *A*_*P*_^*I*^, *A*_*P*_^*O*^ are mutually disjoint set of input, output actions; one denotes by *A*_*P*_ the set of all actions, *A*_*P*_ = *A*_*P*_^*I*^ ∪ *A*_*P*_^*O*^;
4. *T*_*P*_⊆*V*_*P*_ × *A*_*P*_ × *V*_*P*_ is a set of transitions.

**Definition 5**

Set of input/output actions *A*_*P*_^*I*^, *A*_*P*_^*O*^ are described by *a* = (*σ*, *τ*, *λ*), where

1. *σ* is the value of the input/output;
2. *τ* is the time sequence of the input/output;
3. *λ* is the period information of the input/output.

**Definition 6**

A controllable operation segment of the RTEIAP is the alternative finite sequence of states and actions: *v*_0_ *a*_0_ *v*_1_ *a*_1_ *v*_2_ ⋯ *v*_*n*_, where (*v*_*i*_ *a*_*i*_ *v*_*i*+1_) ∈ *T*_*P*_, 0 < *i* ≤ *n*.

One can use an example to explain how a RTEIA describes a system.

**Example 7**

**Figure 2:**
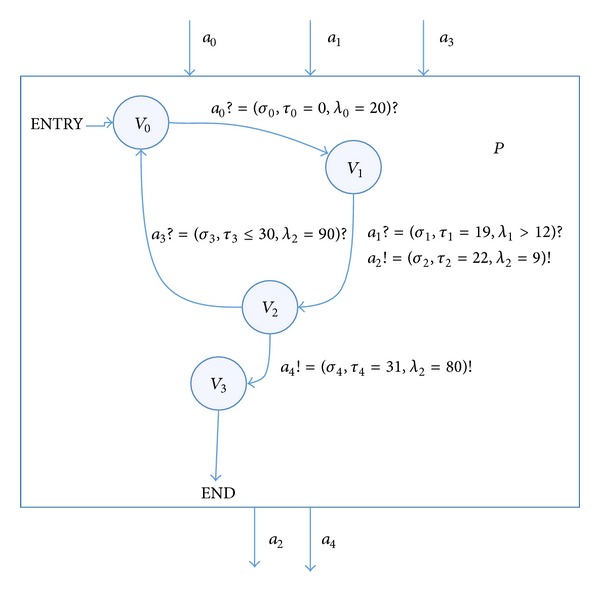
A simple example of the RTEIA *P*.

Figure 2 shows a simple RTEIA, according to the following definition.

RTEIA *P* is as follows.

1. the set of states *V*_*p*_ = {*v*_0_, *v*_1_, *v*_2_, *v*_3_}.
2. the initial set of states *V*_*P*_^init^ = {*v*_0_}.
3. the set of input actions *A*_*P*_^*I*^ = {*a*_0_, *a*_1_, *a*_3_} amd the set of output actions *A*_*P*_^*O*^ = {*a*_2_, *a*_4_}. The input/output actions *a* can be described by time words:
  - *a*_0_ = (*σ*_0_, *τ*_0_ = 0, *λ*_0_ = 20),
  - *a*_1_ = (*σ*_1_, *τ*_1_ = 19, *λ*_1_ = 12),
  - *a*_2_ = (*σ*_2_, *τ*_2_ = 22, *λ*_2_ = 9),
  - *a*_3_ = (*σ*_3_, *τ*_3_ ≤ 30, *λ*_3_ = 90),
  - *a*_4_ = (*σ*_4_, *τ*_4_ = 31, *λ*_2_ = 80).
4. There are a number of transition chains, like *t*_1_ = (*v*_0_ *a*_0_ *v*_1_ *a*_1_ *a*_2_ *v*_2_ *a*_4_ *v*_3_) and *t*_2_ = (*v*_0_ *a*_0_ *v*_1_ *a*_1_ *a*_2_ *v*_2_ *a*_3_ *v*_0_ *a*_0_ *v*_1_ *a*_1_ *a*_2_ *v*_2_ *a*_4_ *v*_3_), *t*_1_, *t*_2_ ∈ *T*_*P*_.

Due to the application of time words to describe the input/output actions, we can choose different transition chain to describe the temporal information of the automaton interface interaction.

### 3.3. Input Control of the RTEIA

For the purpose of applying the RTEIA to software testing, we need to control the input in order to generate test cases. So, we treat the input *a* ^*I*^ as a RTEIA *I*_*i*_. The input automata can cross-link with the device under test and other input source devices, to realize the feedback and control of the input.

**Definition 8**

One defines the influence interface action as a constraint input, denoted by → *a*. In the graphic, dotted arrow is used to denote the constraint input.

**Example 9**

**Figure 3:**
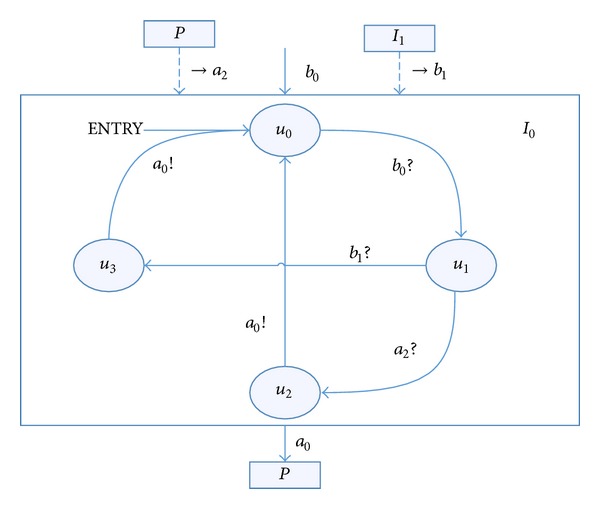
Input automata *I*_0_.

We can establish the RTEIA model for the two input *a*_0_, *a*_1_ from the automata *P* in Example 7, which is shown in Figures 2 and 3.

RTEIA *I*_0_ is as follows.

1. the set of states *V*_*p*_ = {*u*_0_, *u*_1_, *u*_2_, *u*_3_}.
2. the initial set of states *V*_*P*_^init^ = {*u*_0_}.
3. The set of input actions *A*_*P*_^*I*^ = {→*a*_2_, →*b*_1_, *b*_2_} and the set of output actions *A*_*P*_^*O*^ = {*a*_2_}.
4. There are a number of transition chains, like *t*_3_ = (*u*_0_ *b*_0_ *u*_1_ *a*_2_ *u*_2_ *a*_0_ *u*_0_) and *t*_4_ = (*u*_0_ *b*_0_ *u*_1_ *b*_2_ *u*_3_ *a*_0_ *u*_0_), *t*_3_, *t*_4_ ∈ *T*_*P*_.

RTEIA *I*_1_ is as follows.

1. the set of states *V*_*p*_ = {*w*_0_, *w*_1_, *w*_2_}.
2. the initial set of states *V*_*P*_^init^ = {*w*_0_}.
3. the set of input actions *A*_*P*_^*I*^ = {*c*_1_, *c*_2_} and the set of output actions *A*_*P*_^*O*^ = {*a*_1_, *b*_1_}.
4. There are a number of transition chains, like *t*_5_ = (*w*_0_ *c*_1_ *w*_1_ *a*_1_ *w*_0_) and  *t*_6_ = (*u*_0_ *b*_0_ *u*_1_ *b*_2_ *u*_3_ *a*_0_ *u*_0_), *t*_5_, *t*_6_ ∈ *T*_*P*_.

We can find that the feedback of the input *a*_0_ has been described by putting automata *P*'s output *a*_2_ as input automata *I*_0_'s input. Input automata *I*_1_ are also cross-linked with input automata *I*_0_. Furthermore, we can control the input of automata *P* by the design of the internal structure of the input automata *I*_0_ *I*_1_ and the manual adjustment of inputs *b*_0_, *c*_0_, and *c*_1_. For example, in different state transition chains like *t*_3_ and *t*_4_, we can get different *a*_0_ by controlling *a*_2_ and *b*_1_, which are the input of input automata *I*_0_.

There are mainly two characteristics of the RTEIA. Firstly, it uses time words to describe the temporal information (contains both time sequence and period information) of the input/output actions of the automata; secondly, it treat the input interface as independent automata, which is used to control the input by the cross-link of the automata and the input automata.

## 4. Test Cases Generation Based on the RTEIA

Because of the feature of the RTEIA, we apply it to the software testing field. When it is used to describe the software model, we can generate test cases automatically by some algorithms.

### 4.1. Input Automata Controlling

In the interface test of real-time system, we can establish automata model for each input as input automata, using the method introduced in Section 3.3, in order to control the input and realize interface covering by design of the input automata's internal structure.

Common input automata for software interface test are shown in Figure 5.

**Figure 4:**
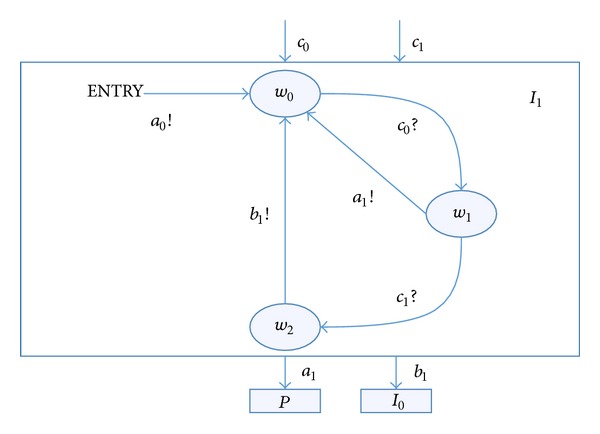
Input automata *I*_1_.

The input automata *I*_0_ are as follows (Figure 4).

1. the set of states *V*_*p*_ = {*u*_0_, *u*_1_, *u*_2_, *u*_3_, *u*_4_, *u*_5_, *u*_6_, *u*_41_, *u*_42_, *u*_51_, *u*_52_, *u*_61_, *u*_62_}.
2. the initial set of states *V*_*P*_^init^ = {*u*_0_}.
3. the set of input actions *A*_*P*_^*I*^ = {→*a*_1_, *b*_0_, *b*_1_, *b*_2_} and the set of output actions *A*_*P*_^*O*^ = {*a*_0_}.
4. There are a number of transition chains, like *t*_1_ = (*u*_0_ *a*_1_ *u*_1_ *b*_0_ *a*_0_) and *t*_2_ = (*u*_0_ *a*_1_ *u*_1_ *b*_0_ *u*_3_ *b*_1_ *u*_5_ *b*_2_ *u*_52_ *a*_0_), *t*_1_, *t*_2_ ∈ *T*_*P*_.

In the input automata, *u*_0_ denotes the initial state of the automata and →*a*_1_ denotes a constraint input (coming from the main automata or another input automata) of the input automata; it means that this input is restricted by the constraint from other part; *u*_1_ denotes the start state. By inputting different *b*_0_, *b*_1_, *b*_2_, we can make the input automata *I* produce normal/abnormal output. *u*_2_ denotes normal state, which will produce a normal test case   *a*_0_, and *u*_4_, *u*_5_, *u*_6_ denote value abnormal state, time sequence abnormal state, and period information abnormal state, respectively. Then, *a*_0_ is produced correspondingly by controlling the input *b*_2_ to make the abnormal value lager or littler. The input automata aim to state all possibilities of the input information, which could be expected or unexpected.

For instance, the state transition chain *t*_2_ = (*u*_0_ *a*_1_ *u*_1_ *b*_0_ *u*_3_ *b*_1_ *u*_5_ *b*_2_ *u*_52_ *a*_0_) denotes a time sequence abnormal *a*_0_, and its time value is too large, which means it will be inputted later than the normal one. The example is just a simple input automata model in logic. We can design a more complex internal structure of the input automata to express different test strategy in practical application.

### 4.2. Test Cases Generation

We simplify the method of software interface covering by the input automata introduced in Section 4.1. When the input automata are finished with the path covering, the interface covering, which means the coverage of the interface status while test cases had been executed, of the software under test is able to be guaranteed too. We present an algorithm to generate test cases for the input automata.

**Definition 10**

A controllable operation segment *Exec*(*v*_0_ *a*_0_ *v*_1_) is a state action (*v*_0_ *a*_0_ *v*_1_) from a state transition chain, where *head*(*Exec*(*v*_0_ *a*_0_ *v*_1_)) = *v*_0_,  *tail*(*Exec*(*v*_0_ *a*_0_ *v*_1_)) = *v*_1_.

We apply a depth-first algorithm to cover these input automata. It will start with the ENTRY. When a controllable operating segment is searched and marked, a new search will start from the tail of the last controllable operating segment. This step will be repeated constantly.

The steps of Algorithm 1 can be summarized as follows.

**Step 1**

When *i* = 1, find a controllable operation segment that *head*(*Exec*(*i*)) = ENTRY.

**Step 2**

If all the controllable operation segments have been found or the coverage has been 100%, then go to Step 6; else, go to Step 4.

**Step 3**

Search for the next unused controllable operation segment which has tail of the last one as the head state; if found, then *i* = *i* + 1 and go to Step 2; else, go to Step 4;

**Step 4**

Let *j* = *i*; record the current sequence number of tested controllable operation segment. Record this operation chain as *Case*(*k*) which starts with ENTRY and ends with END. Run the test and refresh the coverage.

**Step 5**

Search for another controllable operation segment which has the same head state with *Exec*(*i*) and is not marked. If found, *i* = *j* + 1 and go to Step 2; else, *i* = *i* − 1 and repeat Step 5. If all the controllable operation segments have marked, then go to Step 6.

**Step 6**

Display result and finish the algorithm.

We can get all kind of the input time word *a* using this algorithm in order to realize the interface covering of the software under test.

Then, make these time words as the input fields. Choose input groups from the input fields as the input of the testing using some intelligent algorithms such as genetic algorithm or ant colony algorithm. Finally, generate the test cases set by optimizing of the feedback of the coverage continuously aiming at both the interface covering and the path covering.

### 4.3. Case Study

We apply the RTEIA and the test case generation algorithm to an interface test of an aircraft braking system. The structure of the system is shown in Figure 6.

Automata *P* are an aircraft braking system, which has 4 inputs *a*_0_ *a*_2_ *a*_3_ *a*_4_, corresponding to 4 input automata *I*_0_ *I*_1_ *I*_2_ *I*_3_. There are 3 outputs *a*_1_ *a*_5_ *a*_6_; in the same time, *a*_1_ is an input →*a*_1_ of input automata *I*_1_ which functions as a feedback.

Establish the RTEIA model, which is shown in Figure 7.

The meaning of each parameter is shown in Table 1.

The unit of *τ* and *λ* is second and the units of *σ* are dimensionless, Newton, meter per second, round per minute, and millivolt.

Taking an example of the input *a*_2_, establish the input automata *I*_1_, which is shown in Figure 8.

**Figure 5:**
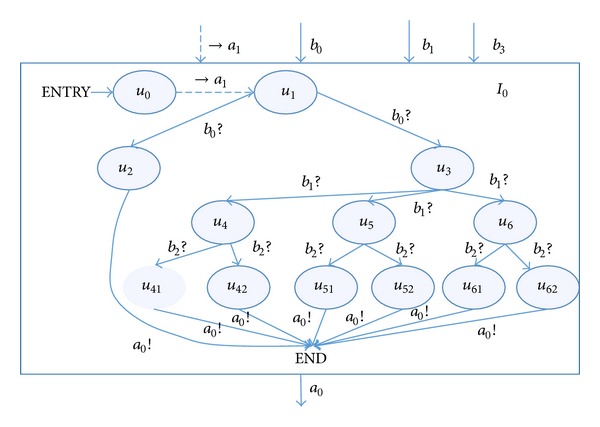
Input automata *I*_0_.

**Figure 6:**
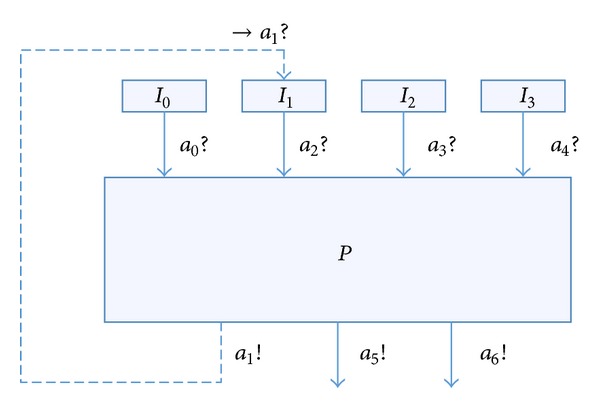
The structure of the aircraft braking system.

**Figure 7:**
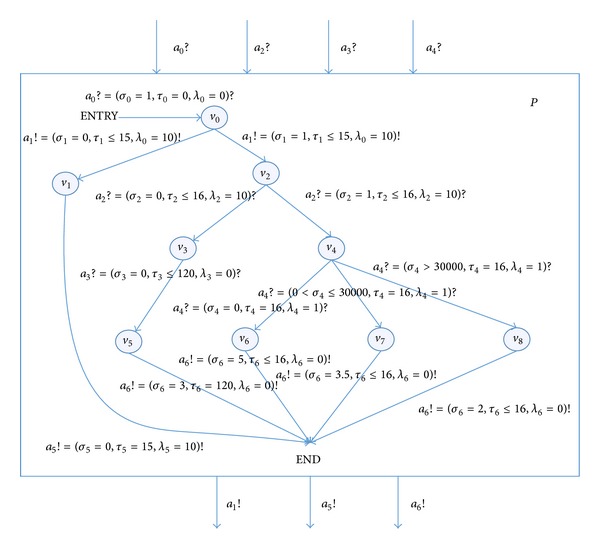
The aircraft braking system automata *P*.

**Figure 8:**
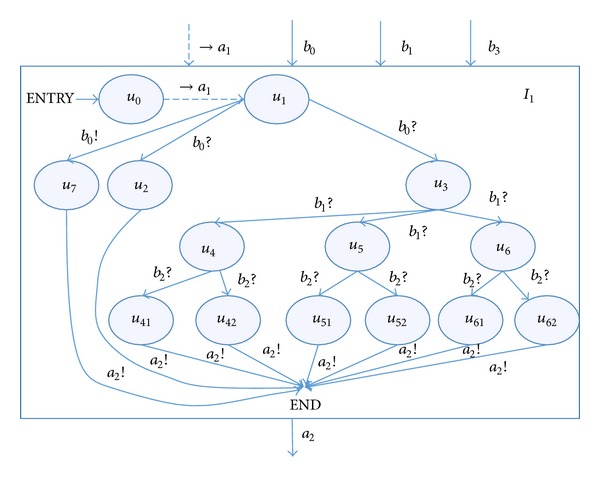
Input automata *I*_1_ of the aircraft braking system.

The meaning of the parameters is similar to Figure 5. The constraint input →*a*_1_ means a feedback that, only when the brake system BIT is successful, can the process begin to work. *u*_2_ *u*_7_ are two kind of normal state and *u*_4_, *u*_5_, *u*_6_ are value abnormal state, time sequence abnormal state, and period information abnormal state, respectively. *b*_2_ controls whether we will get a larger or a littler output *a*_2_.

Using Algorithm 1, we can realize the path covering of the input automata and get a test sequence controlled by *b*_0_ *b*_1_ *b*_2_. By running the test sequence, finally, we are able to get the input field of *a*_2_ as follows:

(4) A2={(σ2=0,τ2=10,λ2=10),    (σ2=1,τ2=10,λ2=10),    (σ2=−36,τ2=10,λ2=10),    (σ2=100,τ2=10,λ2=10),    (σ2=0,τ2=16,λ2=10),    (σ2=1,τ2=17,λ2=10),    (σ2=0,τ2=1,λ2=1),    (σ2=1,τ2=10,λ2=29)}.

Analogously, we can get the input fields of *a*_0_ *a*_3_ *a*_4_:

(5) A0={(σ0=1,τ0=0,λ0=0),    (σ0=−5,τ0=0,λ0=0),    (σ0=32,τ0=0,λ0=0),    (σ0=1,τ0=22,λ0=0),    (σ0=1,τ0=0,λ0=90)},A3={(σ3=0,τ3=100,λ3=0),    (σ3=−9,τ3=100,λ3=0),    (σ3=12,τ3=100,λ3=0),    (σ3=0,τ3=120,λ3=0),    (σ3=0,τ3=178,λ3=0),    (σ3=0,τ3=100,λ3=23)},A4={(σ4=0,τ4=16,λ4=1),    (σ4=20000,τ4=16,λ4=1),    (σ4=43000,τ4=16,λ4=1),    (σ4=30000,τ4=16,λ4=1),    (σ4=0,τ4=10,λ4=1),    (σ4=20000,τ4=10,λ4=1),    (σ4=43000,τ4=10,λ4=1),    (σ4=0,τ4=22,λ4=1),    (σ4=20000,τ4=22,λ4=1),    (σ4=43000,τ4=22,λ4=1),    (σ4=0,τ4=16,λ4=0.5),    (σ4=20000,τ4=16,λ4=0.5),    (σ4=43000,τ4=16,λ4=0.5),    (σ4=0,τ4=16,λ4=8),    (σ4=20000,τ4=16,λ4=8),    (σ4=43000,τ4=16,λ4=8)}.

After getting the input fields of all inputs of the RTEIA, we use some classical intelligent algorithms such as genetic algorithm to reduce the useless cases and to improve the testing efficiency. The sets of input fields are the inputs of the algorithm; the coverage of the automata could be used as the feedback indicator; both the interface covering and the path covering could be selected as the multiple targets of the algorithm. Then, the test set will be got. One typical testing sequence is as follows:

(6) TestCase1=ENTRY→a0?=(σ0=1, τ0=0, λ0=0)?v0 →a1!=(σ1=1, τ1≤15, λ0=10)!v2 →a2?=(σ2=1, τ2=17, λ2=10)?v4 →a4?=(σ4=43000, τ4=16, λ4=8)?v8 →a6!=(σ6=2, τ6≤16, λ6=0)!END.

This testing sequence means that, firstly, the system receives a startup command input *a*_0_, which is a normal input; then the system trans to *v*_0_ which denotes the BIT state, and BIT runs successfully and gets a normal output *a*_1_; then, system receives the landing information input *a*_2_; this input is a time sequence abnormal input; its time is later than the required time in 1 second; then the system trans to state *v*_4_ which denotes the landing state and receives the gear wheel speed input *a*_4_; the speed is 43000 which belongs to the fast landing. This input is a period information abnormal input; its period is amplified to 8; then the system trans to state *v*_8_, outputs signal *a*_6_ of the gear hydraulic circuit to reduce the braking friction strength and prevent the gear locking; then the system ends.

By using the RTEIA to describe the interface testing of this above real-time system with temporal information accurately, testing cases have been generated automatically to improve the testing efficiency successfully.

## 5. Discussions

In terms of the functioning features, compared with the related interface automata model, such as the interface automata, the timed automata, and the timed interface automata, because of the introducing and improving of the time words, the RTEIA makes the description of temporal information more complete; besides, for establishing input automata, the RTEIA is more powerful when it is applied in the software testing field. The contrast is shown in Table 2.

The RTEIA has superiority in the description of the temporal information and input control in the field of software testing. In terms of the description of temporal information, the interface automata do not contain temporal information; it is difficult to describe periodic signal for the timed automata and the timed interface automata, though there is some improvement. In terms of the judgment of combination and complexity, because the RTEIA concentrates on the field of software testing, it shows different features from the traditional methods. In terms of the input control, unlike the traditional methods, the RTEIA treats every input as independent automata, by which it can control the input conveniently. In terms of the application in software testing field, the RTEIA is more suitable than some other related researches about interface automaton and timed interface automata because of their limited temporal description, the interface covering guarantee, and so forth.

In terms of the algorithmic complexity, input interface automata can be represented by a directed graph. The main cost of Algorithm 1 is in the searching for the controllable operation segments corresponding to the distinguishable states. Therefore, if the interface automata are expressed in the form of adjacency matrix, the algorithm complexity would be *O*(*n* ^2^), where *n* denotes the state number of the longest execution segments; if the interface automata are expressed in the form of adjacency list, the algorithm complexity would be *O*(*n* + *m*), where *m* denotes the number of the execution segments.

The RTEIA solved the problems of traditional automata modeling language successfully without any increase in cost.

## 6. Conclusions

This paper proposes a new formalized modeling language named the real-time extended interface automata to describe the temporal information of the interface between the system and the environment. Based on the interface automata, the RTEIA solves the problems of the traditional automata methods by introducing time words to describe the temporal information (including the period information) of input/output actions. Meanwhile, the RTEIA is able to control the input conveniently and to realize both the path and interface covering when applied in software testing field by establishing the input automata. At last, we provided relevant algorithm and process and verified its feasibility and effectiveness by applying this method in the testing of one real aircraft braking system.

Because the RTEIA is a new method, there are still some open questions left to be solved. As part of the future work, we will investigate the ways to describe the feedback and the constraints between the input automata, and so forth. Besides, the RTEIA will be tried to be applied in more cases and fields.

## Figures and Tables

**Algorithm 1:**
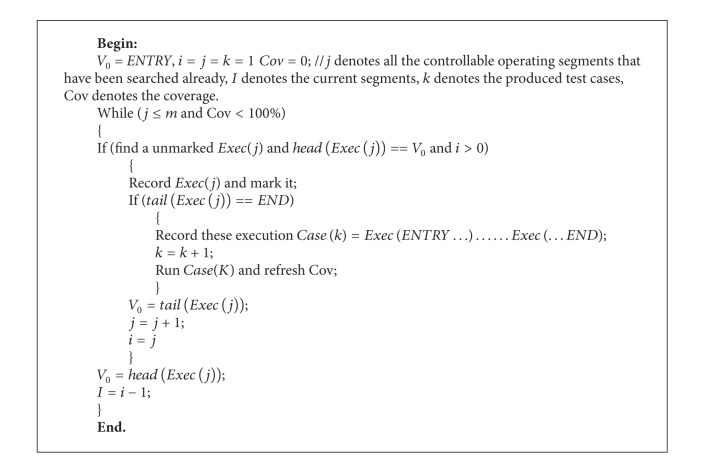


**Table 1:** Parameters of the aircraft braking system.

Parameter	Meaning
*v*_0_	State of system BIT
*v*_1_	State of a failed system BIT
*v*_2_	State of a successful system BIT, system startup
*v*_3_	State of taking off
*v*_4_	State of landing
*v*_5_	State of stop turning braking: the braking system is used to completely stop the idling of the landing gear tires after taking off
*v*_6_	State of keeping in land: the braking system is used to lock the landing gear tires after landing
*v*_7_	State of normal braking
*v*_8_	State of antilock braking
*a*_0_	Input: command of the brake system startup
*a*_1_	Output: the result of the system BIT
*a*_2_	Input: the information of takeoff/landing
*a*_3_	Input: the gear's load information, to determine whether the plane has completely off the ground during takeoff
*a*_4_	Input: the gear wheel speed information, to determine when the plane landed at rest, a normal speed, or a too fast speed
*a*_5_	Output: problem report of the system BIT
*a*_6_	Output: the gear hydraulic circuit signal, to control the force created by the hydraulic institute and implement different braking friction strength.

**Table 2:** Comparing RTEIA with other automata models.

Model type	Description of temporal information	Judgment of combination and complexity	Input control	Being applied in the software testing field
Interface automata	No	Yes	No	Yes, focuses on path covering and does not contain temporal information
Timed automata	Yes, can describe time information by logic relationship	No	No	Yes, focuses on collating logic information and path covering
Timed interface automata	Yes, but cannot describe period information	Yes	No	No
RTEIA	Yes, can describe temporal information by time words	No	Yes, can control the input by establishing the input automata	Yes, can describe temporal information completely and contain interface covering.
